# Acute haemodynamic effects of dobutamine in experimental sepsis-induced myocardial depression

**DOI:** 10.1186/2197-425X-3-S1-A799

**Published:** 2015-10-01

**Authors:** DT Andreis, W Khaliq, M Singer

**Affiliations:** University College London, Bloomsbury Institute of Intensive Care Medicine, London, United Kingdom; Università degli Studi di Milano, Dipartimento di Fisiopatologia Medico-Chirurgica e dei Trapianti, Milan, Italy

## Introduction

Septic patients with myocardial depression are routinely treated with dobutamine[1]. Whether this strategy is desirable is questionable, as catecholamines increase cardiac work, reduce myocardial efficiency, and are cardiotoxic[2]. We can accurately predict mortality in a 72-hour fluid-resuscitated rat model of faecal peritonitis as early as 6 hours, based on the degree of myocardial depression (low stroke volume, high heart rate)[3]. This model offers a useful means of testing safety and efficacy of therapeutic interventions in predicted survivors and non-survivors.

## Objectives

To compare dose-related haemodynamic effects of dobutamine at 6 hours post-insult in predicted survivors and non-survivors from faecal peritonitis.

## Methods

Male Wistar rats (341 ± 33 g) were instrumented with arterial and central venous lines. Sepsis was induced (ip injection of faecal slurry), and fluid resuscitation (10 ml/kg/h) started 2 hours later. At 6 hours, animals were assigned to good prognosis or poor prognosis groups - depending on echo-derived stroke volume (cutoff value 0.20 ml, based on previous experiments). An additional fluid bolus (10 ml/kg) was given, followed by dobutamine infusion, increasing from 5 to 20 µg/kg/min in 2.5-µg/kg/min increments every 5 minutes, with haemodynamic measures recorded just prior. Repeated measures ANOVA and post-hoc Holm-Sidak test were used to seek statistically significant. differences.

## Results

**Figure 1:**
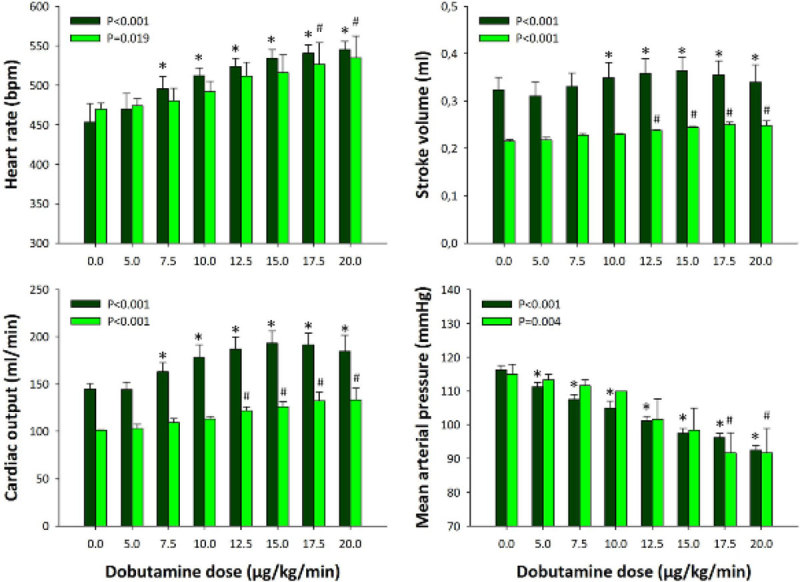
*p < 0.05 ***vs.*** **baseline (good prognosis group, dark blue bars, N = 4).** ^**#**^ **p < 0.05** ***vs.*** **baseline (poor prognosis group, light blue bars, N = 3).**

Stroke volume at 6 h was significantly lower in poor prognosis animals; good prognosis animals were more responsive than poor prognosis animals to dobutamine, with earlier rises in heart rate, stroke volume and cardiac output, and a fall in blood pressure (Figure 1).

## Conclusions

The early hypodynamic circulatory profile of poor prognosis septic rats is associated with catecholamine-hyporesponsiveness. This supports an underlying mechanism of impaired adrenergic signal transduction, and/or dysfunctional downstream pathways. Our data support the investigation of alternative agents for sepsis-induced myocardial depression.

## Grant Acknowledgement

ESICM Basic Science Award, UK Intensive Care Society Young Investigator Award, NIHR.
